# Ultrasensitive Materials for Electrochemical Biosensor Labels

**DOI:** 10.3390/s21010089

**Published:** 2020-12-25

**Authors:** Aneesh Koyappayil, Min-Ho Lee

**Affiliations:** School of Integrative Engineering, Chung-Ang University, 84 Heuseok-ro, Dongjak-Gu, Seoul 06974, Korea; aneeshkdhamodaran@gmail.com

**Keywords:** nanoparticles labels, enzyme labels, redox probe labels, quantum dots labels, low dimensional carbon material labels

## Abstract

Since the fabrication of the first electrochemical biosensor by Leland C. Clark in 1956, various labeled and label-free sensors have been reported for the detection of biomolecules. Labels such as nanoparticles, enzymes, Quantum dots, redox-active molecules, low dimensional carbon materials, etc. have been employed for the detection of biomolecules. Because of the absence of cross-reaction and highly selective detection, labeled biosensors are advantageous and preferred over label-free biosensors. The biosensors with labels depend mainly on optical, magnetic, electrical, and mechanical principles. Labels combined with electrochemical techniques resulted in the selective and sensitive determination of biomolecules. The present review focuses on categorizing the advancement and advantages of different labeling methods applied simultaneously with the electrochemical techniques in the past few decades.

## 1. Introduction

**Scheme 1:**
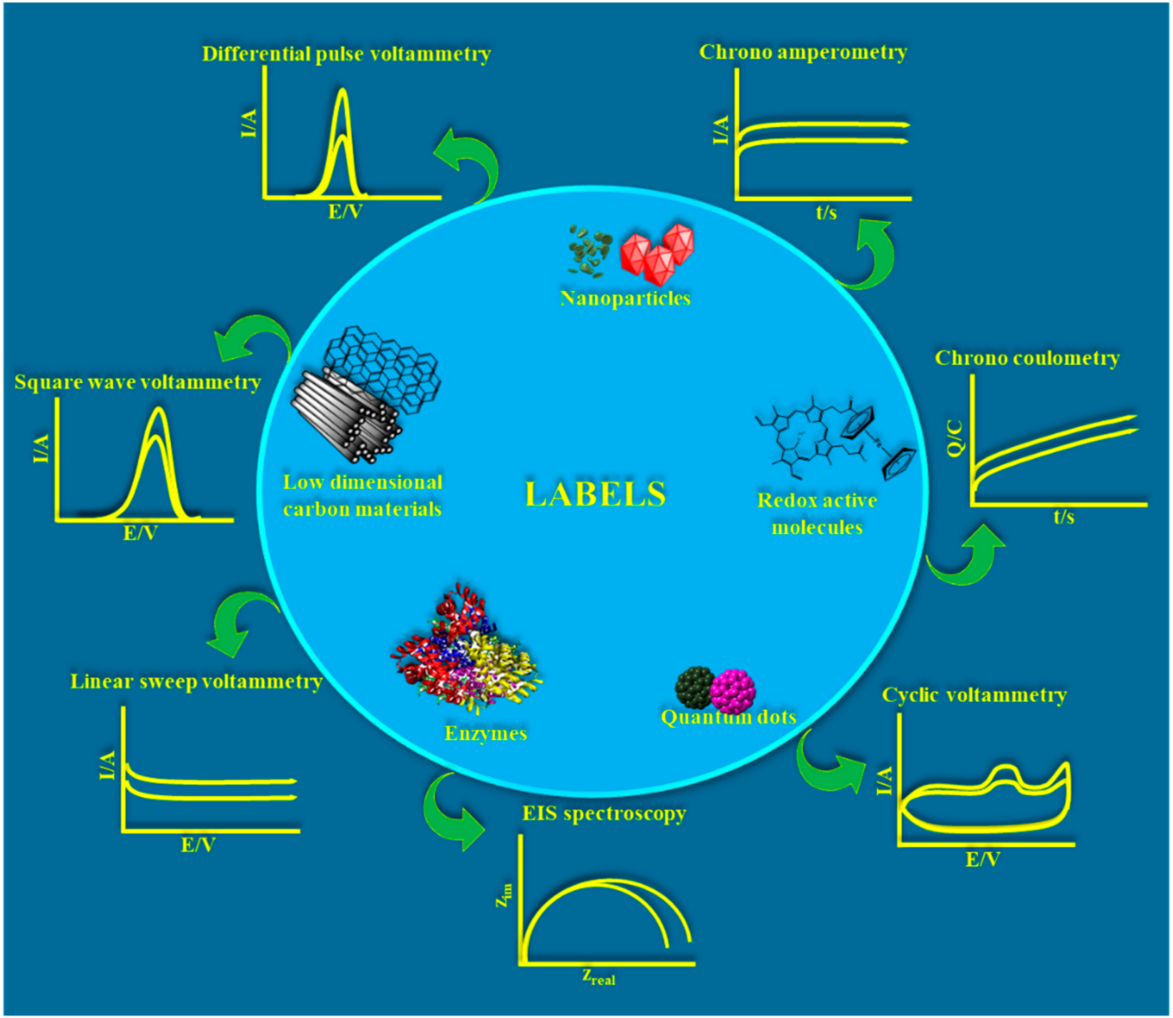
Schematic illustration of various labels combined with electrochemical techniques.

The never-ending demand for improved selectivity and sensitivity has prompted intense research and proposed improved methods for the detection/determination of different analytes in the fields of clinical diagnostics [1,2,3]. Different bioassays based on the specific interaction between the biological recognition elements, such as antibody, nucleotide, enzyme, and the target analyte have been proposed and developed to meet these requirements. These methods are often characterized by low cost, rapid results, high selectivity and specificity, the potential for multiplexed detection/determination, operational and instrumental simplicity, and the scope for point-of-care (POC) applications. In bioassays, the signal is generated from the specific interaction between the analyte molecule and the recognition element, which is usually immobilized on a solid surface, and is used to detect and quantify the analyte by using a suitable transducer. The attractive features of electrochemical transducers are widely adopted in bioassays because of the sensitivity, potential for miniaturization and automation, and simple/often portable instrumentation. The advantages of label-free sensors such as less complicated designs, less preparation time, reduced cost due to the elimination of complex labels, and scalability are severely overwhelmed by the disadvantages such as lack of sensitivity, cross-reactivity, and interference. Labeled sensors are preferred over label-free designs when adequate preparation facilities and trained personnel are available. Labels are combined with techniques such as optical [4], electrochemical [5], fluorescence [6], Raman technique [7], magnetic [8], electro chemiluminescent [9], etc., for the selective and sensitive detection of analytes. Labels combined with electrochemical techniques have gained considerable momentum over the past decades because of the advancements in electrochemical techniques [10,11,12,13,14,15,16,17]. Electrochemical labels based on the principle of bio-specificity interactions have gained attention in clinical diagnosis or environmental monitoring because of the sensitivity, simplicity, low cost of analysis, and miniaturization possibilities [18]. Labels combined with electrochemical techniques generate a current response suitable for the detection/determination of analytes with high selectivity and specificity. Despite the increasing number of published works and interest in labels in electrochemical biosensing, there is a lack of reviews covering specifically the application of labels with electrochemistry, and the results are often uncategorized. In this context, this review aims to categorize different labels and present a general overview of the recent applications and advancements of labels in electrochemical sensors (Scheme 1).

## 2. Enzyme Labels Combined with Electrochemical Applications

**Figure 1:**
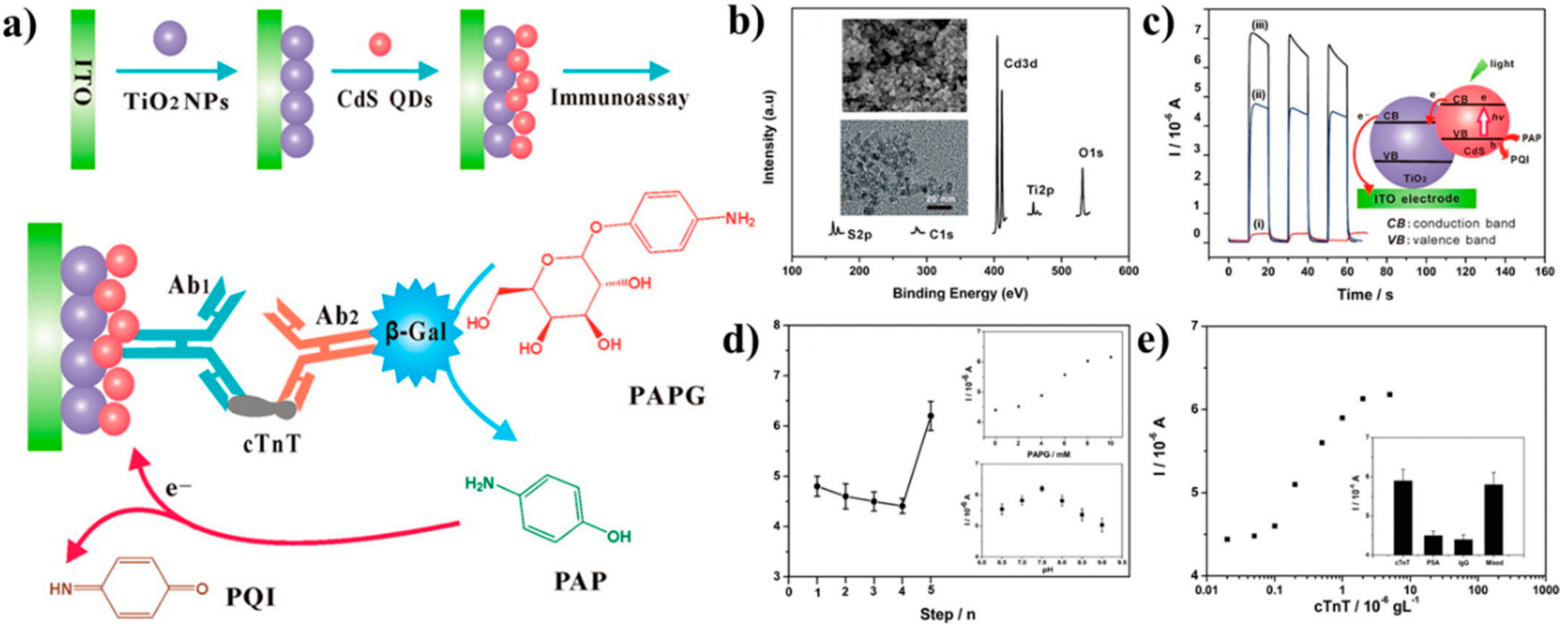
(**a**) Schematics of the photoelectrochemical immunoassay for the detection of cTnT using the β- galactosidase tags. (**b**) XPS spectrum of the CdS QDs/TiO_2_ NPs electrode (the inset above shows the SEM image of the TiO_2_ NPs film and below shows the TEM image of the CdS QDs). (**c**) Photoelectrochemical responses of the TiO_2_ NPs electrode (i), after loading with the CdS QDs (ii), and in the presence of 10 mM PAP (iii). (**d**) Photocurrent response from the CdS QDs/TiO_2_ NPs electrode (step 1), the electrode after immobilization with Ab1 and BSA blocking (step 2), after target recognition (step 3), after complexing with Ab2 (step 4), and after incubation in 10 mM PAPG (step 5) (the inset above shows the effect of PAPG on the photocurrent intensity in the presence of 1.0 × 10^−6^ g/mL of cTnT, and the inset below shows the photoelectrochemical response as a function of pH). (**e**) Photocurrent of the immunoassay in the presence of different cTnT concentrations (the inset shows the selectivity of the sensor in the presence of PSA, IgG, and the mixed sample). Reproduced with permission from [21] Copyright © 2020, American Chemical Society.

Enzymatic catalytic properties are renowned for the determination of extremely small quantities of reactants, thereby making these natural catalysts an efficient label. Enzyme labels have advantages such as the possibility of simultaneous assay because of the availability of a wide variety of enzymes, availability of different conjugation techniques, sensitive assays due to the amplification effect of enzymes, and the availability of cheap reagents for cost-effective assays [19]. Enzyme labels with the advancement in electrochemical techniques resulted in selective, sensitive, and rapid assays. Enzyme labels such as Alkaline phosphatase (ALP), Horseradish peroxidase (HRP), Glucose oxidase (GOx), Glucose-6-phosphate dehydrogenase (G6PDH), β-galactosidase (β-gal), and DT-diaphorase (DT-D) are combined with detection systems such as electrochemical, colorimetric, fluorimetry, and chemiluminescent for the detection of target analytes. An efficient enzyme label should catalyze the enzymatic reaction on its substrate at a sufficiently high reaction rate and exhibit long-term stability. These requirements are satisfied by enzymes such as ALP and HRP, making them the most common enzyme labels [20]. Zhao et al. [21] reported an electrochemical detection system based on photocurrent measurement for the determination of cardiac troponin T(cTnT) using beta-galactosidase as a label. Here, the photocurrent, which was directly correlated to the target analyte, was generated from the photooxidation of p-aminophenol, which, in-turn, was generated from the β-Gal enzyme-catalyzed conversion of p-aminophenyl galactopyranoside (Figure 1). The method is renowned for its importance as the first PEC immunoassay towards the cardiac biomarker.

**Figure 2:**
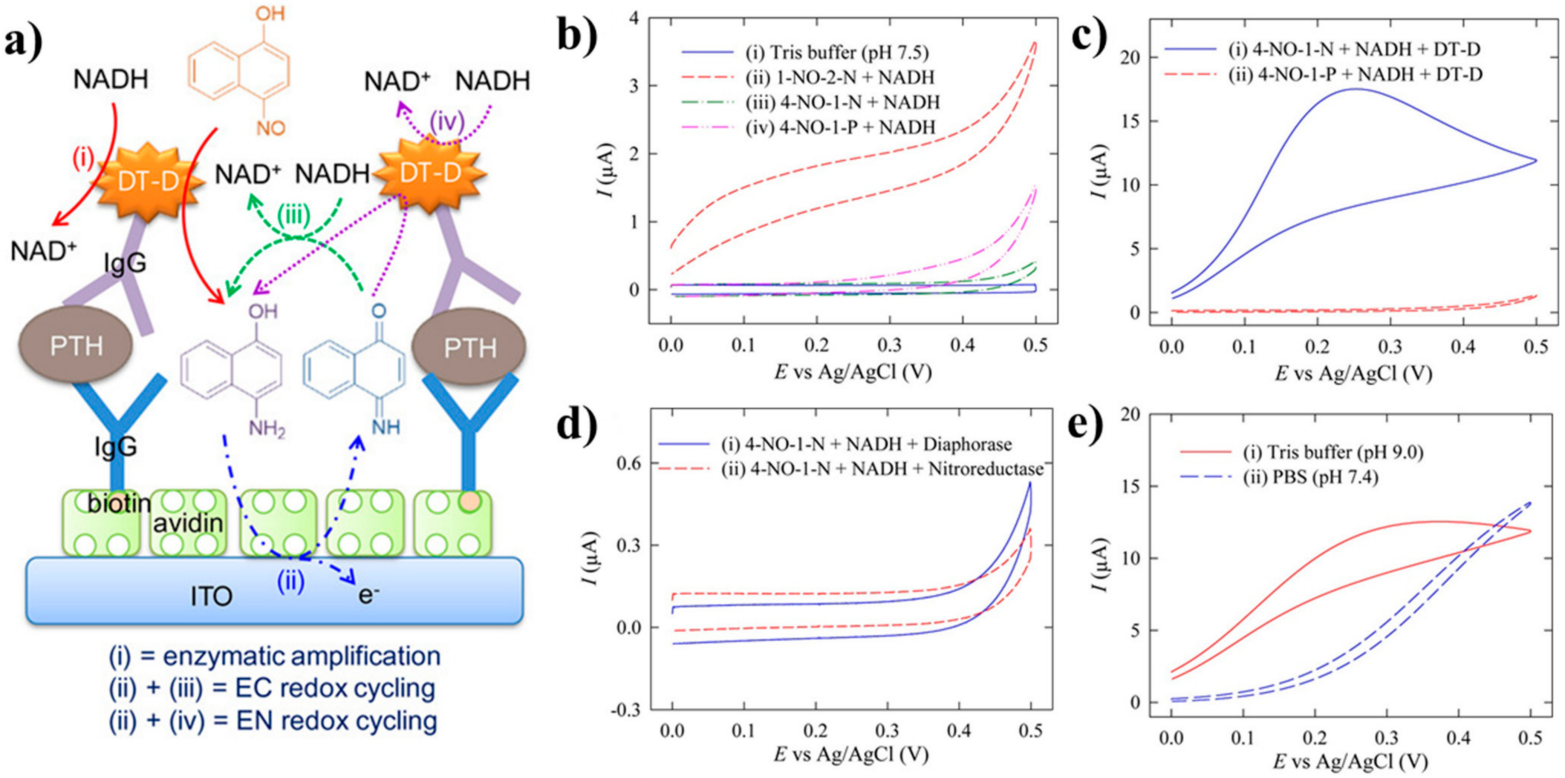
(**a**) Schematics of the electrochemical immunosensor using DT-diaphorase as an enzyme label. (**b**) Cyclic voltammograms (CVs) obtained at the bare indium tin oxide (ITO) electrodes in (i) tris buffer of pH 7.5, (ii) mixture of 1-nitroso-2-naphthol and NADH, (iii) mixture of 4-nitroso-1-naphthol and NADH, (iv) mixture of 4-nitrosophenol and NADH at a scan rate of 20 mV/s after 10 min. incubation period at 25 °C. (**c**) CVs obtained at the bare ITO electrodes in (i) tris buffer of pH 7.5 containing NADH, 4-nitroso-1-naphthol, and DT-diaphorase, (ii) tris buffer of pH 7.5 containing NADH, 4-nitrosophenol, and DT-diaphorase. (**d**) CVs obtained at the bare ITO electrodes in (i) tris buffer of pH 7.5 containing NADH, Diaphorase, and 4-nitroso-1-naphthol, (ii) tris buffer of pH 7.5 containing NADH, nitroreductase, and 4-nitroso-1-naphthol. (**e**) CVs obtained at the bare ITO electrodes in (i) tris buffer of pH 9.0 containing NADH, 4-nitroso-1-naphthol, and DT-diaphorase, and (ii) PBS of pH 7.4 containing 4-nitroso-1-naphthol, NADH, and DT-diaphorase. Reproduced with permission from reference [22]. Copyright © 2020 American Chemical Society.

An interesting enzyme label based on the two-electron reductase enzyme DT-diaphorase (NAD(P)H: Quinone Oxidoreductase1) combined with electrochemical immunosensor application was reported by Kang et al. [22]. Here, the thermostable DT-diaphorase enzyme and an electrochemically inactive compound 4-nitroso-1-naphthol were used as an enzyme label and reacting substrate, respectively. Signal amplification was achieved by the enzymatic amplification and an electrochemical redox process. The electrochemically inactive substrate was converted to an amine compound by the DT-diaphorase, which further undergoes electrochemical-chemical and electrochemical-enzymatic redox process, respectively (Figure 2). The enzyme label-based electrochemical immunosensor was applied for the determination of parathyroid hormone (PTH) over a wide range of concentrations from PBS containing BSA with an LOD of 2 pg/mL for PTH.

## 3. Nanoparticles as Labels for Electrochemical Applications

The inherent instability and other shortcomings associated with enzyme labels have promoted the use of nanoparticles as their substituent. Nanoparticles have many advantages over natural enzymes. The redox current generated from the redox properties enables them to be used as an easy label without any additional steps or reactions in electrochemical immunosensor applications. Some of the well-explored nanoparticle labels include Au NPs [23] and Ag NPs [24], whereas nanoparticle labels such as multimetallic nanoparticles [25], metal oxide nanoparticles [26,27], and metal sulfide nanoparticles [28] have also been explored with interesting applications.

**Figure 3:**
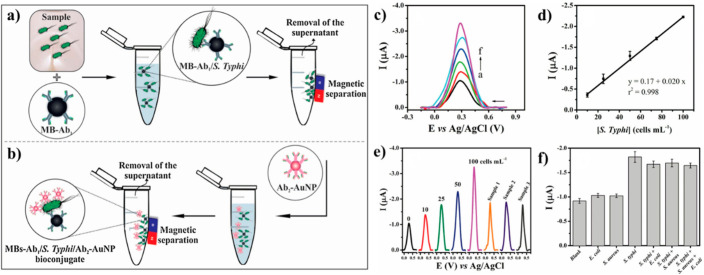
(**a**) Schematic illustration showing the steps involved in the offline *Salmonella typhimurium* capture with MB-AB1 followed by washing. (**b**) The formation of MB-Ab1/S. typhi/Ab2-AuNP magneto-immunoconjugate after the addition of Ab2-AuNP. (**c**) DPV curves for the S. typhi detection (0–100 cells/mL of bacteria) in PBS-T20 (pH 7.4). (**d**) The corresponding calibration curve (n = 8). (**e**) DPV curves obtained for the determination of *Salmonella typhimurium* (0–100 cells/mL) from milk sample using a single DμFD. (**f**) Specificity study of the immunoassay, including negative control (concentration of 25 cells/mL for each bacteria). Reproduced with permission from reference [33]. Copyright © 2020 Elsevier B.V.

Gold particles of nano dimension have attracted attention because of the stability [29], ease of conjugation with biomolecules [30], affinity towards thiol (-SH_2_) group [31], and the shape- and size-related quantum effects [32]. Au NPs serve as an ideal label for electrochemical assay for biomolecules. Various ultrasensitive assays are reported by using Au NPs as the label. Oliveira et al. [33] reported an ultrasensitive magnetoimmuno-assay for the determination of *Salmonella typhimurium* by using a low-cost, disposable microfluidic device (DμFD). Here, the *Salmonella typhimurium* was separated from the samples by capturing with a monoclonal anti-Salmonella antibody modified magnetic beads (Figure 3). This was followed by the addition of Au NPs labeled polyclonal anti-Salmonella antibody to form a magneto-immunoconjugate, which was then injected onto the microfluidic device and captured by placing magnets behind the working electrodes. Finally, the electrochemical response from the labeled Au NPs was used for the determination of the *Salmonella typhimurium*.

**Figure 4:**
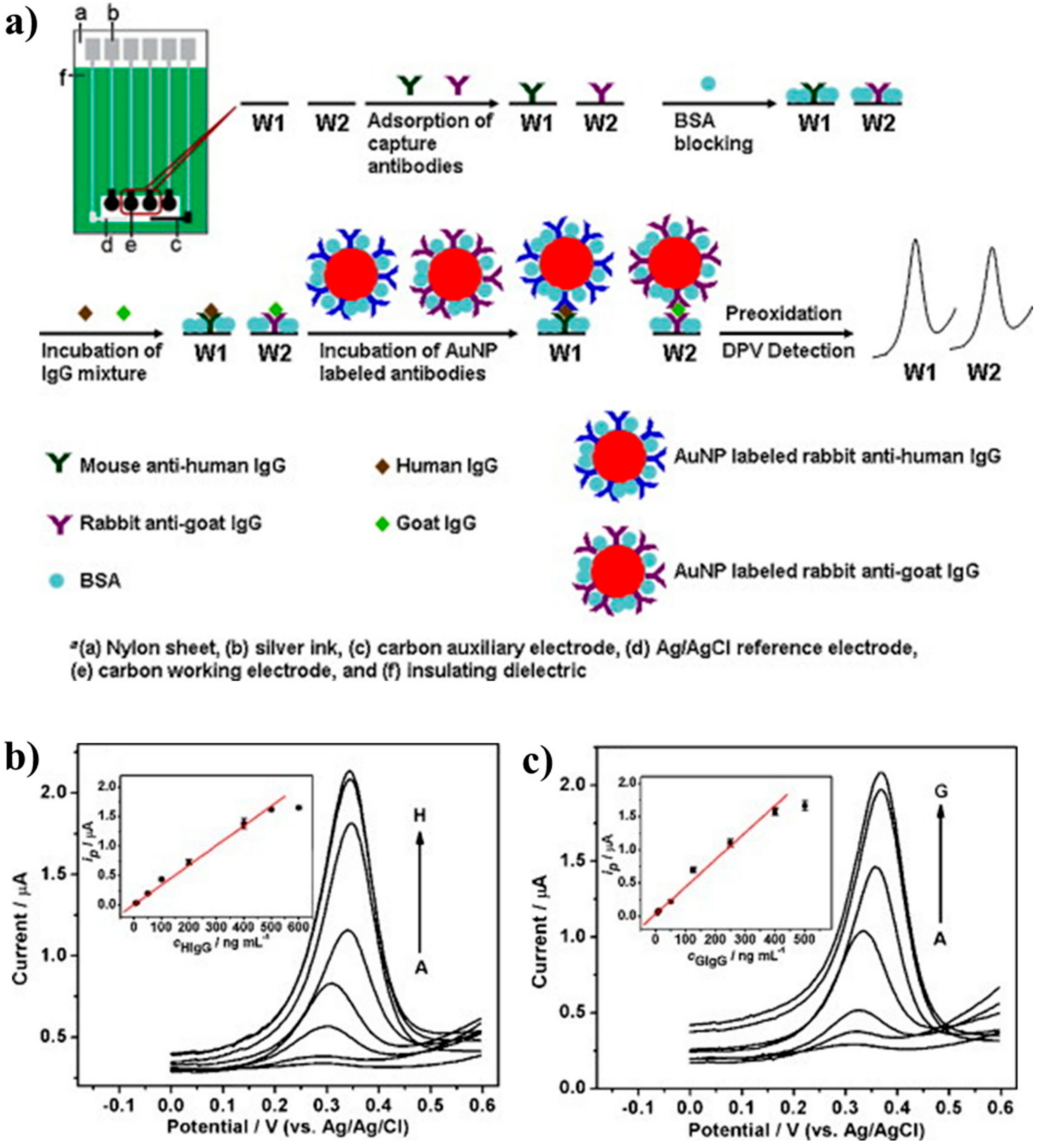
(**a**) Schematics of the preparation of immunosensor array for the simultaneous detection of HIgG and GIgG. (**b**) DPV responses for the simultaneous multiplexed detection of HIgG (5–600 ng/mL), and (**c**) GIgG (5–500 ng/mL) using Au NPs as a label. Insets show the corresponding calibration curves. Reproduced with permission from reference [36]. Copyright © 2020 Elsevier B.V.

Liao et al. [34] reported an interesting strategy for the determination of goat anti-rabbit IgG by using Au NPs as label. Here, the autocatalytic decomposition of Au ions onto the Au NPs and the resulting enlarged Au NPs resulted in a three times amplification of the electrochemical assay compared to Au NPs in the conventional assay. Mao et al. [35] reported an assay for the determination of HIgG by using Au NPs as the label. Here, a chemical reaction between avidin and dethiobiotin in the presence of the vitamin biotin resulted in the cyclic accumulation of Au NPs, and the resultant signal was used for the quantification of IgG up to 0.1 ng/mL. Another interesting example of Au NPs as the label for electrochemical immunoassay was reported by Leng et al. [36]. Here, the capture of Au NPs labeled antibodies and a sandwich format on a disposable chip resulted in the crosstalk-free simultaneous determination of HIgG and GIgG (Figure 4).

**Figure 5:**
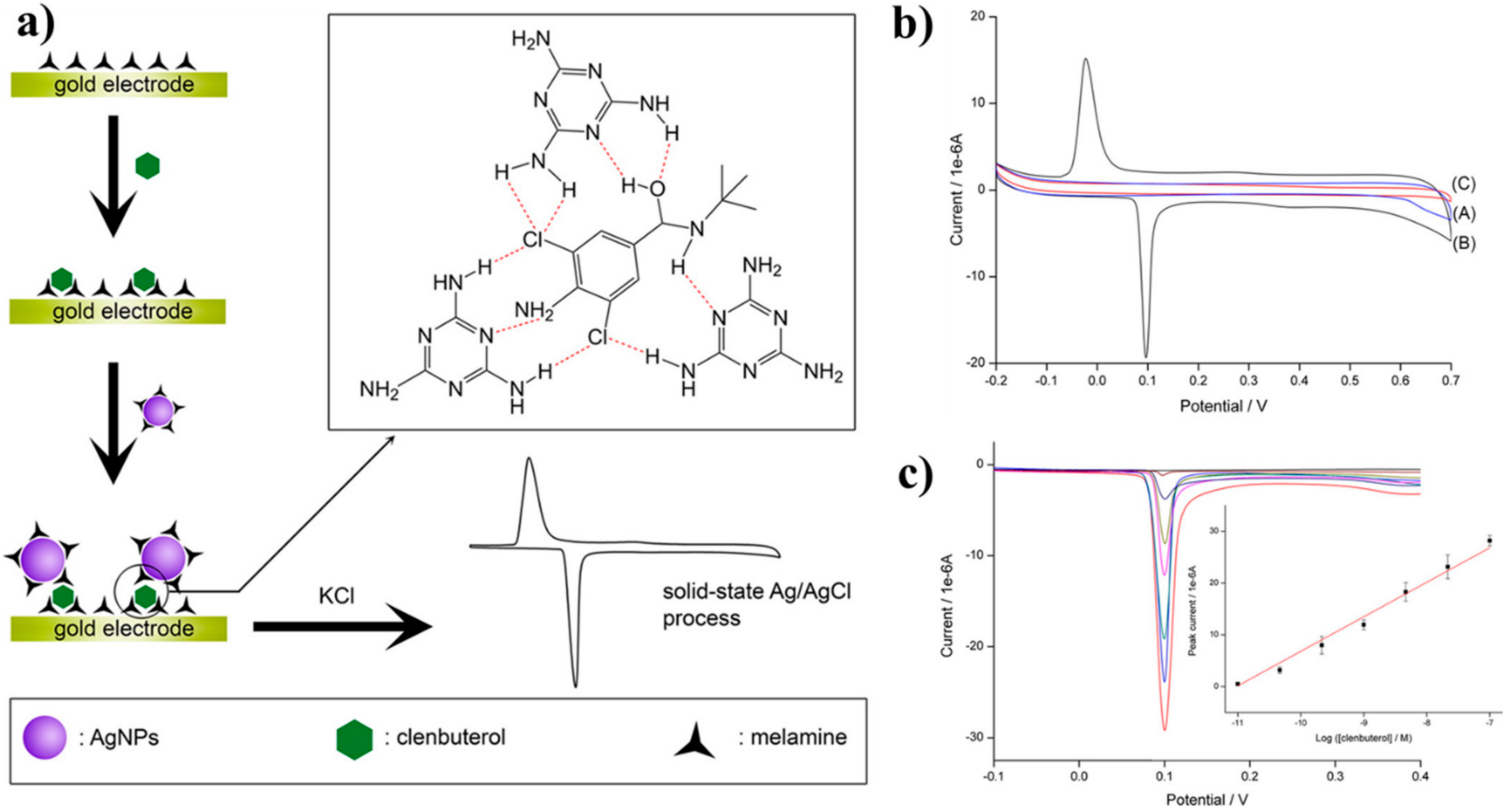
(**a**) Schematics of the melamine-silver nanoparticles (M-Ag NPs)-based sensor for clenbuterol determination. (**b**) CV response of the sensor in the presence of (A) 0, and (B) 1 nM clenbuterol. (C) melamine modified Au electrode at a scan rate of 100 mV/s in the presence of 0.1 M KCl. (**c**) The LSV response of the sensor in the presence of different concentrations of clenbuterol (0–100 nM). Inset shows the corresponding linear calibration plot (n = 3). Reproduced with permission from [24]. Copyright © 2020 American Chemical Society.

The excellent electroactivity combined with well-defined sharp voltammetric oxidation peak makes Ag NPs an excellent choice for electroanalytical applications [37]. In one report, Ag tags were employed for the detection of PSA at fg/mL levels [38]. Miao et al. [24] reported melamine functionalized Ag nanoparticles as label for the determination of important therapeutic agent clenbuterol (Figure 5). Here, the solid-state reaction of Ag/AgCl resulted in sharp, well-defined silver stripping peaks, and an LOD of 10 pM for clenbuterol was achieved.

## 4. Quantum Dots (QDs) as Labels for Electrochemical Applications

**Figure 6:**
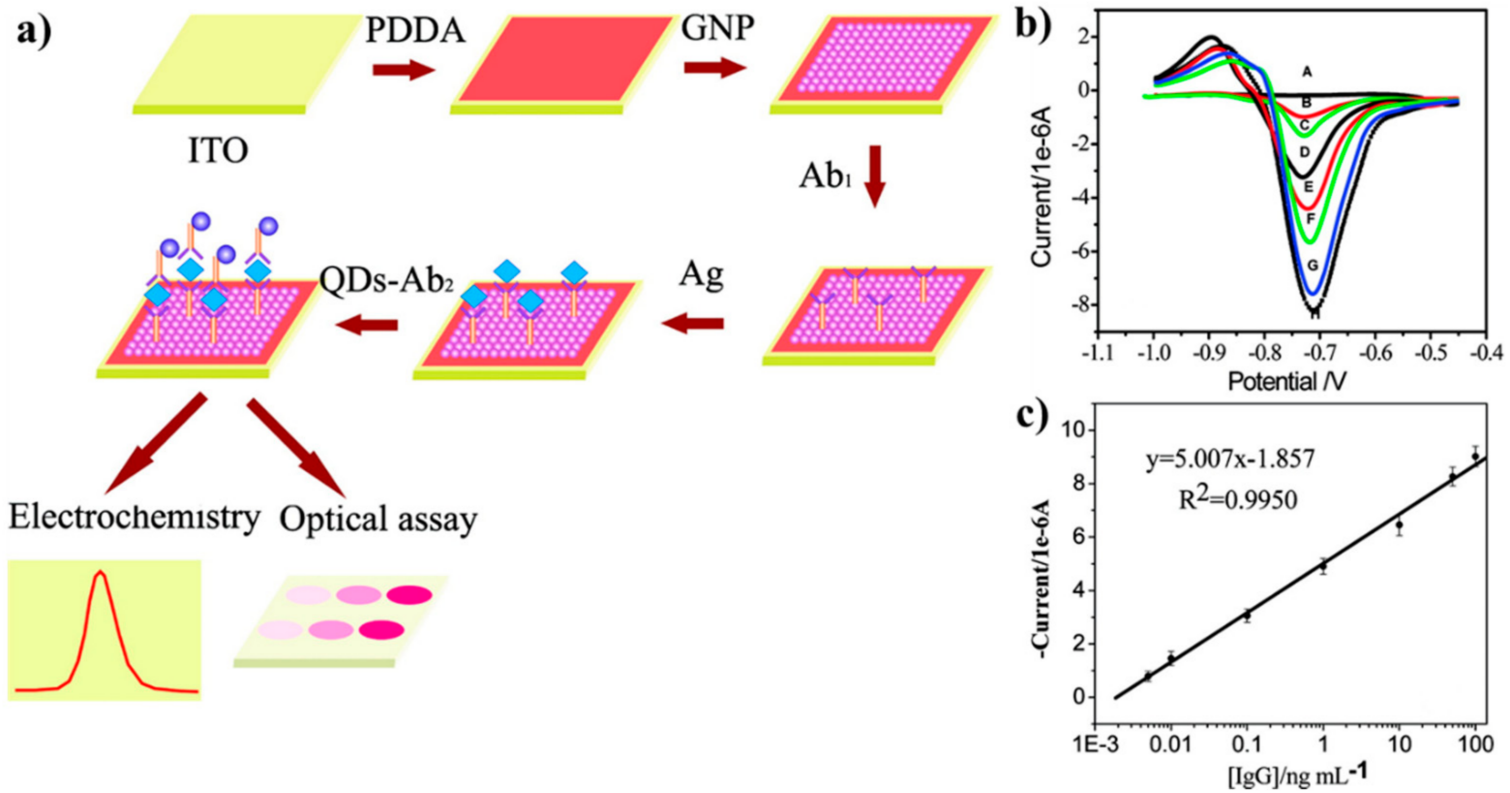
(**a**) Schematics of the CdTe QDs label-based immunoassay for the detection of protein. (**b**) Square wave voltammograms for the determination of HIgG (0–100 ng/mL). (**c**) The corresponding calibration curve on a semilog scale. Reproduced with permission from [46]. Copyright © 2020, American Chemical Society.

QDs are nanostructured and zero-dimensional materials defined as semiconductor structures [39]. Besides the properties of semiconductor metal oxides, QDs exhibit photochemical [40], magnetic [41], optoelectrical [42], and catalytic properties [43]. Since the demonstration of water-soluble and biocompatible QDs [44,45], research on the synthesis of QDs for biosensing applications has gained considerable momentum, and many QDs, such as CdS and CdTe, have been reported and used as labels for electrochemical bio-sensing applications. An ultrasensitive immunoassay for protein HIgG by using CdTe QDs as an electrochemical label was reported by Cui et al. [46]. The method demonstrated a sensitive assay with results consistent in comparison with ELISA (Figure 6).

**Figure 7:**
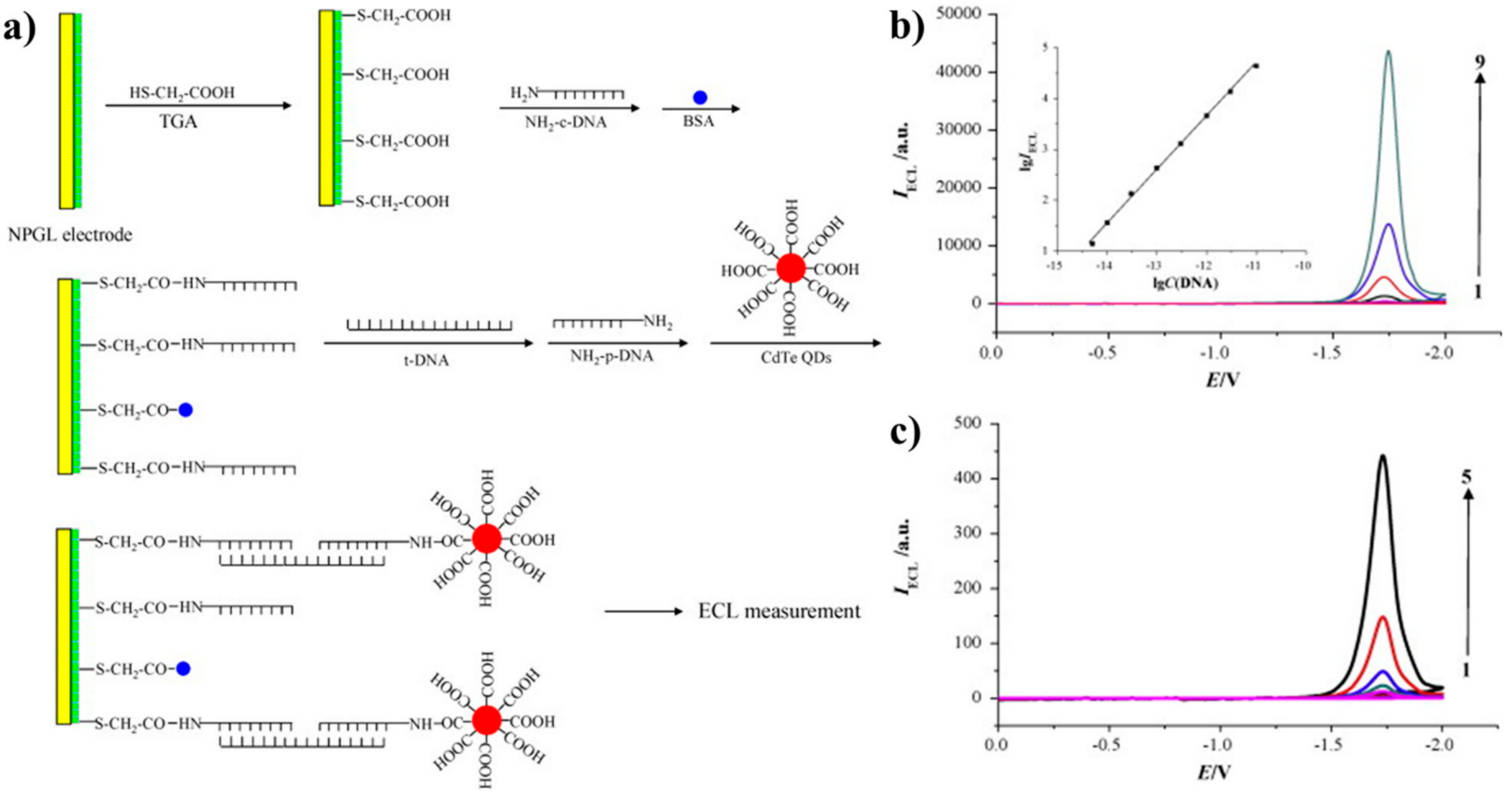
(**a**) Schematics of the process of DNA determination. (**b**) Electro chemiluminescent current (I_ECL_) vs. potential (E) curves for the CdTe Quantum dot-labeled DNA hybrids immobilized on the nanoporous gold leaf electrode for different t-DNA concentrations (the inset shows the linear relationship between I_m,ECL_ on the IECL-E curves and the t-DNA concentration). (**c**). A magnified portion of the I_ECL_-E curve. Reproduced with permission from [47]. Copyright © 2020 Elsevier B.V.

Hu et al. reported CdTe QDs as DNA labels on nanoporous gold leaf electrodes (NPGL) for the ultrasensitive DNA analysis by using electrochemiluminescence as the electrochemical technique (Figure 7). Here, the remarkably increased sensitivity of the assay was attributed to the ultra-thin nanoporous nature of the electrode. The NPGL electrode was first modified with thioglycolic acid (TGA) which was then conjugated to amino-modified c-DNA. Following that, the hybridization of probe DNA with t-DNA yielded sandwich-type hybrids on the nanoporous electrode. The amino end group of the sandwich hybrids was then labeled with mercaptopropionic acid capped CdTe QDs. Finally, the ECL emission of the CdTe QD-labeled electrode was measured by scanning between 0 to −2 V in the presence of peroxydisulfate ion (S_2_O_8_^2−^) as a co-reactant. The ECL intensity recorded was correlated to t-DNA concentration in the linear range of 5 × 10^−15^ − 1 × 10^−11^ M. The study also proposes the possibility of using the ECL DNA assay protocol for the determination of mRNA in cell extracts [47].

**Figure 8:**
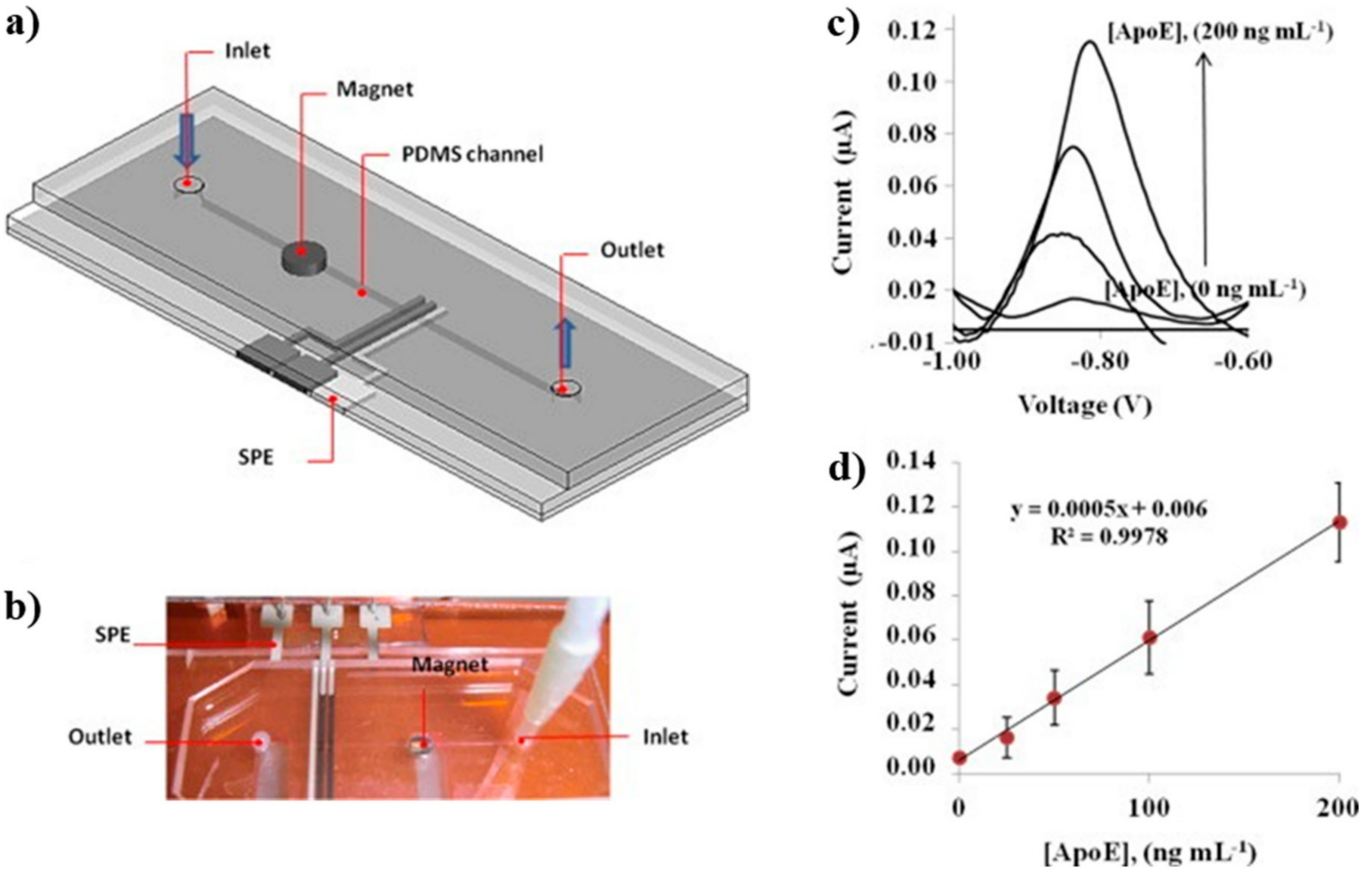
(**a**) Schematics of the electrochemical immunosensing system for ApoE detection. (**b**) Microfluidic platform integrated with screen–printed electrodes, and the picture of actual the set–up. (**c**) Square wave voltammograms of the ApoE–immunoassay in the presence of different concentrations of ApoE (0–200 ng/mL) using QDs as electrochemical labels (Electrochemical conditions; −1.1 V of deposition potential for 120 s; stripping between −1.1–−0.15 V; V scan rate = 10 mV/s; Frequency = 25 Hz; and flow rate = 5 μL/min). (**d**) The calibration plot for the standard sample of ApoE. Reproduced with permission from [48]. Copyright © 2020 Elsevier B.V.

Sánchez et al. proposed a cadmium-selenide/zinc-sulfide (CdSe@ZnS) quantum dots (QDs)-based on-chip magneto-immunoassay for the SWASV-based electrochemical detection of Alzheimer’s biomarker ApoE [48]. The electrochemical sensing platform was established by integrating screen-printed electrodes on to a hybrid polydimethylsiloxane-polycarbonate microfluidic chip with tosyl activated magnetic beads as a preconcentration platform (Figure 8). The ApoE was quantified with a limit of detection of ~12.5 ng/mL with a linear range of 10–200 ng/mL. The method was successful for the accurate determination of ApoE from diluted human plasma.

## 5. Redox-Active Molecules as Labels for Electrochemical Applications

**Figure 9:**
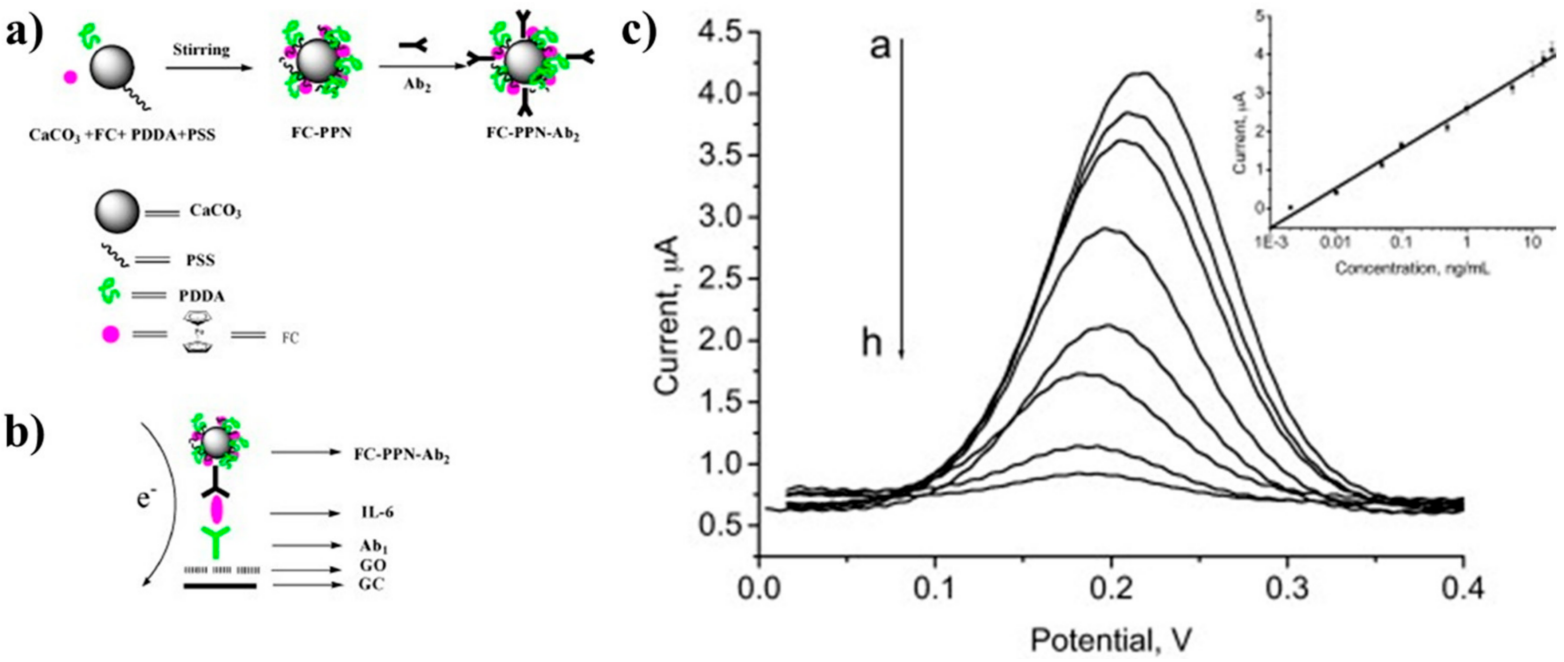
(**a**) Schematics of the fabrication of ferrocene loaded porous polyelectrolyte nanoparticles–Ab2 and (**b**) the immunosensor. (**c**) SWV response of the immunosensor towards different concentrations of interleukin–6 (0.01–20 ng/mL). Scanning the potential window was from 0–0.4 V with a frequency of 25 Hz using a 5–mV potential step and 25 mV amplitude. The insert is the corresponding calibration curve. Reproduced with permission from reference [49]. Copyright © 2020 Elsevier B.V.

**Figure 10:**
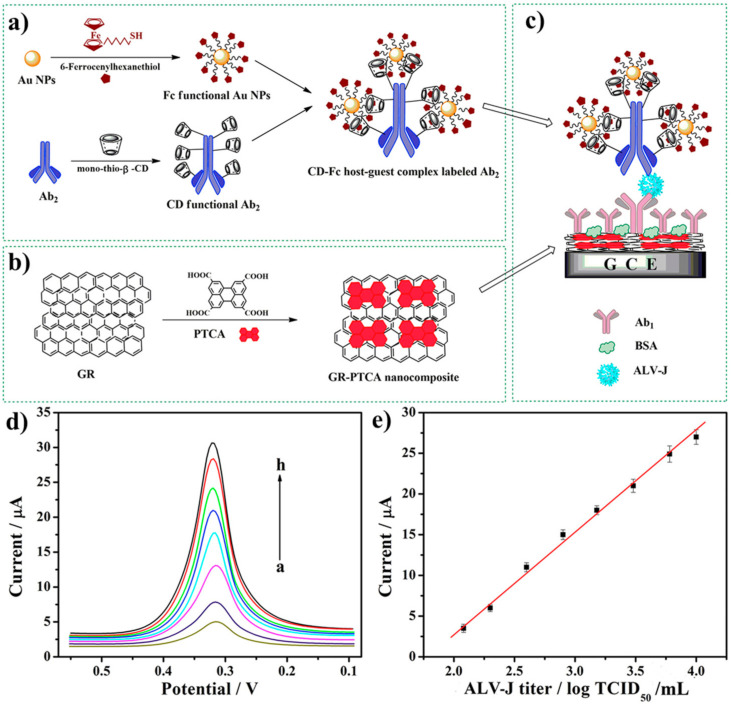
(**a**) Schematics of the fabrication of Ferrocene–AuNP–β–cyclodextrin–Ab_2_, (**b**) GR–PTCA, and (**c**) the electrochemical immunosensor. (**d**) The DPV responses of the immunosensor in the presence of varying concentrations of avian leukosis virus (10^2.0–^10^4.0^TCID_50_/mL). (**e**) The linear calibration plot for the determination of avian leukosis virus. Reproduced with permission from reference [50]. Copyright © 2020 Elsevier B.V.

The redox current from redox probes can serve as an excellent label for electrochemical applications. Li et al. [49] reported ferrocene-loaded polyelectrolyte nanoparticles on the CaCO_3_ template as a label for the detection of oral cancer biomarker IL-6. The ferrocene redox current from square wave voltammetry was exploited for the selective and sensitive determination of interleukin-6 with a wide linear range of 0.002–20 ng/mL, and a low detection limit of 1 pg/mL (Figure 9). Ning et al. [50] reported an ultrasensitive electrochemical sensor for avian leukosis virus-based on differential pulse voltammetry using β-cyclodextrin-nanogold-ferrocene host-guest label on a perylene-3,4,9,10-tetracarboxylic acid-functionalized graphene composite (GR-PTCA). The abundance of polycarboxylic sites on GR-PTCA and the excellent electrical conductivity was suitable for the immobilization of primary antibodies and promoted electron transfer. As shown in Figure 10, signal amplification was achieved by the strong binding of β-cyclodextrin with ferrocene-gold nanoparticles. The sensor achieved linearity for the determination of avian leukosis virus in the concentration range of 10^2.0–^10^4.0^ TCID_50_/mL, and a low detection limit of 10^1.93^ TCID_50_/mL. Mohammadniaei et al. [5] reported an interesting redox-active molecule labeled electrochemical biosensor and signal amplification strategy based on Mxene and Au NPs. Here, the combination of methylene blue and ferrocene labeled single-stranded DNAs on Au NPs decorated Mxene nanosheets resulted in the rapid and multiple quantifications of microRNAs. The Mxene decorated with Au NPs provided a four-time electrochemical signal enhancement compared to Au NPs/Au electrode due to the surface area enhancement and charge mobility increment of the electrode. The method was successfully applied for the attomolar detection of miR-21 and miR-141 with a wide linear range (500 aM–50 nM).

## 6. Low Dimensional Carbon Materials as Ultrasensitive Labels

**Figure 11:**
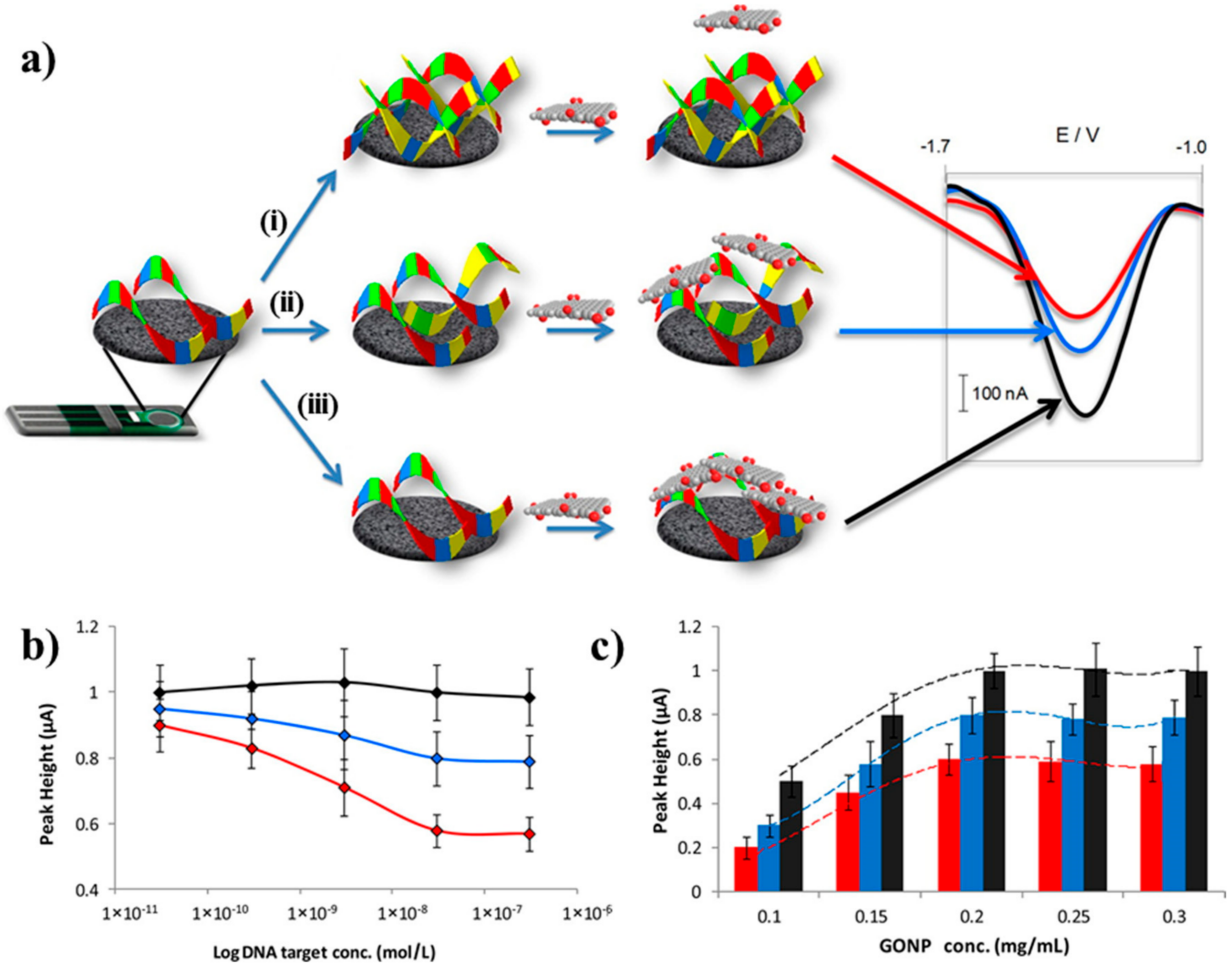
(**a**) Schematics of the experimental protocol, (i) after complementary target hybridization, (ii) one–mismatch target, and (iii) non–complementary target. (**b**) Voltammetric response towards different target DNAs in the case of hybridization with wild–type (red line), mutant (blue line), and non–complementary (black line). (**c**) Voltammetric signal for conjugation with different amounts of GO NPs in the case of hybridization with wild–type (red bars), mutant (blue bars), and nc (black bars) sequences (n = 3). Reproduced with permission from Ref. [63]. Copyright (2012) American Chemical Society.

Graphene and graphene-related materials are renowned for exceptional electrical, chemical, thermal, mechanical, and optical properties [51,52,53,54,55]. The significant interest of graphene and related materials in electrochemical applications is mainly due to the large surface area and the fast charge transfer kinetics [56,57,58,59,60,61]. These unique features also make them promising signaling labels for bio-recognition events in biosensing applications. Graphene and related materials can serve as a “Direct label” or “Indirect label” for signal generation in immunoassays [62]. The direct labeling strategy exploits the working signal generated from the reduction of the oxygen-containing groups, whereas the indirect label strategy relies on the graphene labels modified with other electroactive probes for the generation of working signal [57]. Bonanni et al. [63] reported graphene oxide as a direct label for the detection of DNA polymorphism (Figure 11). Here, the DNA probes were immobilized on a carbon electrode followed by hybridization with a DNA sequence showing single nucleotide polymorphism, and a non-complementary sequence was used as the negative control. The hybrid modified electrodes were able to conjugate to different amounts of graphene oxide because of the differential affinity of graphene towards single and double-strand DNA. The reduction signal generated from the conjugated hybrid-electrode were able to discriminate the single nucleotide polymorphism at 10 nM level in the analyzed sequences.

**Figure 12:**
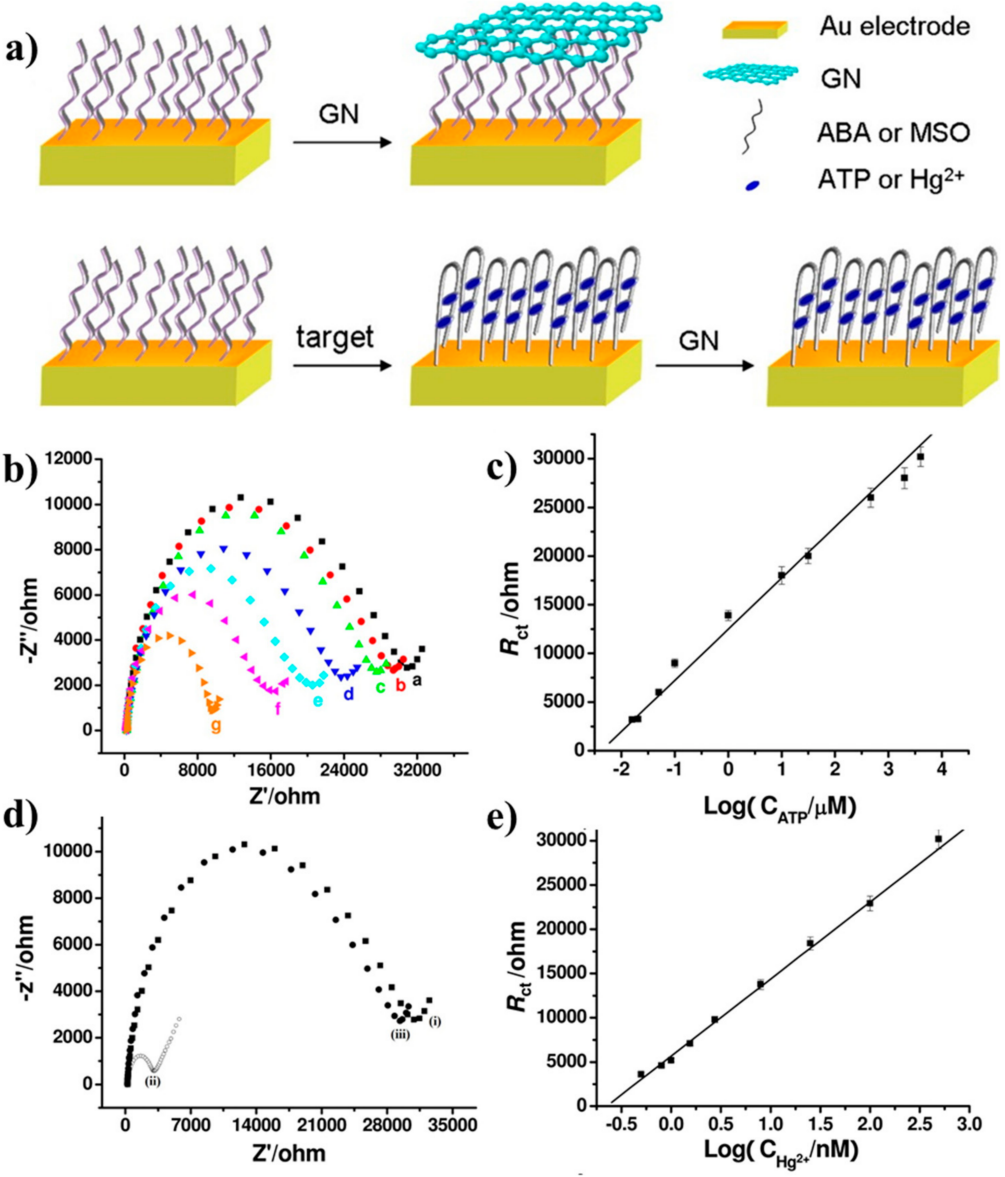
(**a**) Schematics of the sensing platform for the detection of ATP. (**b**) EIS response on the ABA/Au electrode after reacting with different concentrations of ATP (4 mM–0.1 μM) for 1 h and incubation with 0.3 mg/mL GN. (**c**) The corresponding relationship between R_ct_ and ATP concentration (n = 3). (**d**) EIS response on (i) MSO/Au film, (ii) MSO/Au film incubated with GN, and (iii) MSO/Au reacted with 800 nM Hg^2+^ and then incubated with the GN. (**e**) The linear relationship between the EIS and the concentration of Hg^2+^. Reproduced with permission from reference [64]. Copyright © 2020 American Chemical Society.

In the indirect labeling strategy, the working signal is not directly generated from the graphene. Here, an electroactive species was added during the detection process and the graphene label serves to promote the transfer of electrons. An interesting example is the impedimetric determination of adenosine triphosphate (ATP) reported by Wang et al. [64]. Here, a decrease in the charge transfer resistance (Rct) of the system was observed when an ATP-aptamer modified Au electrode was adsorbed with graphene (Figure 12). The aptamer-analyte complex formed in the presence of ATP prevented the graphene interaction with ATP-aptamer resulted in a higher Rct.

**Figure 13:**
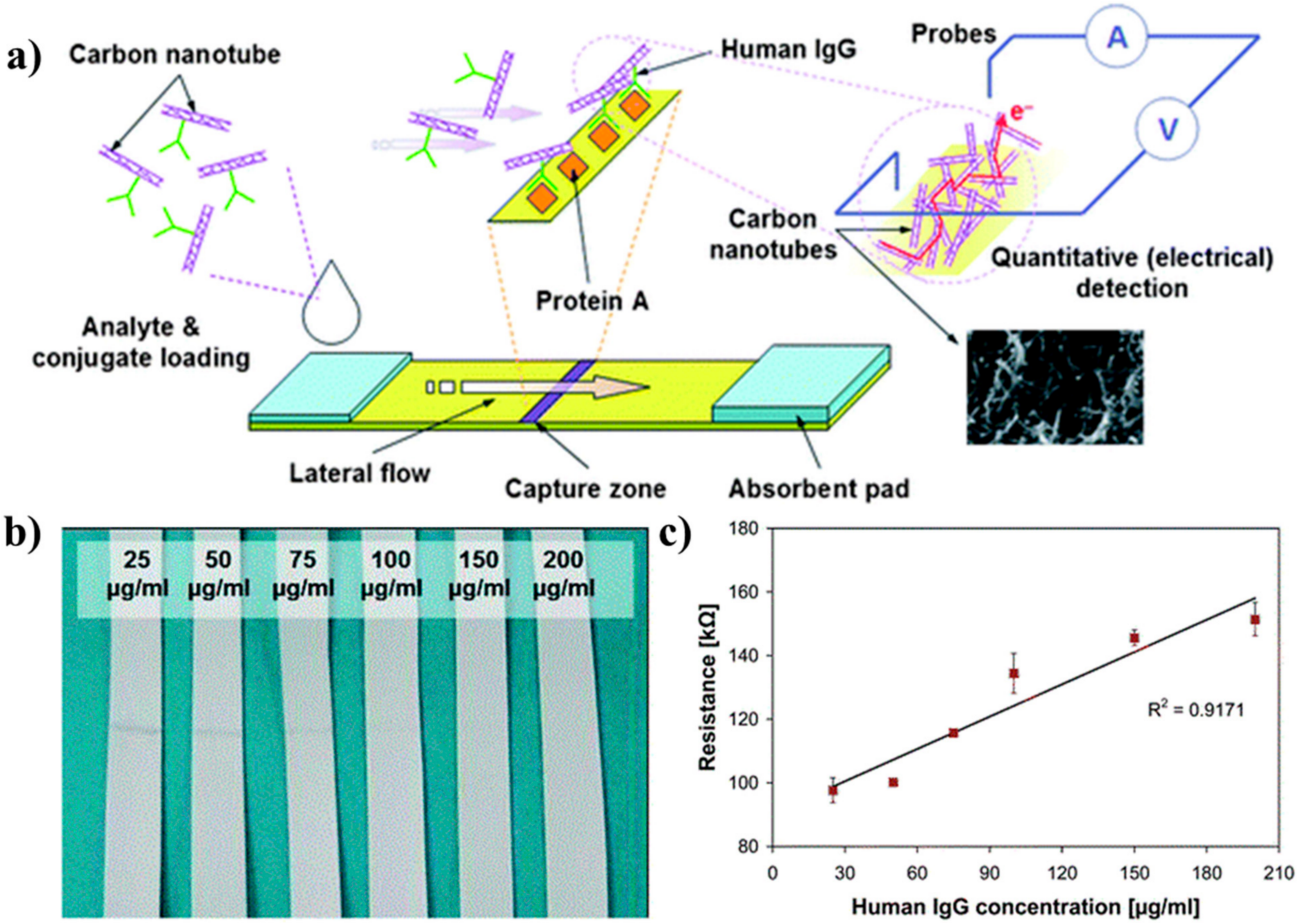
(**a**) Schematics of the quantitative lateral flow immunoassay using CNTs as label. (**b**) The lateral flow immunosensor (7 mm width) with varying concentrations of HIgG. (**c**) The plot of the electrical resistance at the capture zones vs. HIgG concentration. Reproduced with permission from reference [68]. Copyright © 2020 Royal Society of Chemistry.

The remarkable one–dimension conductance [65] and the possibility of specific biomolecule adsorption [66,67] on the surface have prompted the application of CNTs as an efficient label for biosensing applications. Adeyabeba Abera and Jin–Woo Choi [68] reported a lateral flow–based novel immune assay based on MWCNTs as a label for the determination of HIgG. Here, the HIgG was first immobilized on the surface of bare MWCNTs and applied on a lateral flow immunosensor (Figure 13). The MWCNT–HIgG conjugate formed a conducting network, which was then captured at the capture zone by an immobilized protein. The conductivity at the capture zone varied with the amount of HIgG binding with MWCNT and a simple resistance measurement quantified the HIgG. The method was able to quantify HIgG in the range of 25–200 μg/mL without the requirement for an amplification step.

Even though the correct choice of the label and the electrochemical technique is to be determined from the application perspective, labels combined with suitable electrochemical techniques often result in sensitive assays helps overcome various challenges such as low detection limit and high sensitivity. Table 1 summarizes the various categories of electroactive materials (redox-activity or electrocatalytic activity towards secondary reactions) reported as labels in electrochemical applications. It is noteworthy that sensitive assays resulting from the combination of labels and electrochemical techniques are advantageous for POC devices and real sample analysis.

## 7. Summary and Outlook

With the advancement in electrochemical techniques, various ultrasensitive materials such as enzymes, nanoparticles, redox-active molecules, quantum dots, low dimensional carbon materials, etc. have been reported as labels for the sensitive and selective determinations of target analytes. This review focuses on the advancement and advantages of various labels combined with electrochemical techniques to meet the high requirements for biomedical applications over the past few decades. Several fabrication methods and novel functionalization methods are discussed, while some interesting techniques are emphasized in detail in this review. Each of these labels has advantages and drawbacks that should be balanced with the needs of a specific application. By analyzing the design of various types of labels, the trend of designing new multifunctional labels can be pointed out. However, numerous obstacles, such as the reproducibility and scope for massive production, remain for the commercial utilization of these ultrasensitive labeled sensors. More systematic studies involving the optimization of different labeling protocols are still needed.

## Figures and Tables

**Table 1:** Literature comparison of the analytical performance of various labels reported for electrochemical applications.

Label Type	Label	Electrochemical Technique	Target	Linear Range	LOD	Ref.
Enzymes	β-gal	PEC	cTnT	----	1.0 × 10^−7^ g/L	[21]
	DT-D	Chronocoulometry	PTH	2 pg/mL - 1 μg/mL	2 pg/mL	[22]
	GOx	PEC	α-SYN	50 pg/mL - 100 ng/mL	34 pg/mL	[69]
	ALP	PEC	PSA	----	0.5 ng/mL	[70]
	LOx	Amperometry	CA125	0.01–100U/mL	0.002 U/mL	[71]
	HRP	Amperometry	PCB	0.1–50 μg/mL	0.1 μg/mL	[72]
	GPDH	Chronocoulometry	cTnI	----	10 pg/mL	[73]
Nanoparticles	Ag NPs	LSV	Clenbuterol	0.01–100 nM	0.01 nM	[24]
	ZnS NPs	DPV	Codeine	73 pM - 73 nM	37 pM	[28]
	Au NPs	SWSV	DNA	0.52–1300 aM	0.35 aM	[74]
	Au-Ag NPs	DPV	*E. coli*	----	10^2^ CFU/mL	[25]
	Pt NPs	LSV	Adenosine	1–750 nM	1 nM	[75]
	Pd NPs	Amperometry	AFP	0.1–50,000 pg/mL	0.033 pg/mL	[76]
	Cu NPs	DPV	GSH	1–1000 nM	0.27 nM	[77]
	Ir NPs	Amperometry	CEA	0.5–5000 pg/mL	0.23 pg/mL	[78]
	IrO_2_ NPs	Amperometry	PDBE	----	21.5 ppb	[26]
	Fe_3_O_4_ NPs	PEC	PSA	0.05–1000 pg/mL	18 fg/mL	[27]
	MSNs	Amperometry	HIgG	0.01–10 ng/mL	5 pg/mL	[79]
	MoS_2_ NPs	Amperometry	RIgG	----	1.94 pg/mL	[80]
Quantum dots	CdS QDs	SWV	PSA	0.005–10 ng/mL	3 pg/mL	[81]
	CdTe QDs	SWV	HIgG	0.005–100 ng/mL	5 pg/mL	[46]
	CdSe@ZnS QDs	DPV	anti-tTG IgG	0–40 U/mL	2.2 U/mL	[82]
Redox-active molecules	Hemin	DPV	DNA	----	2.35 ng/mL	[83]
	Methylene blue	DPV	SARS DNA	1–25 μM	800 nM	[84]
	Ru(bpy)_3_^2+^	ECL	CEA	0.2–2000 μg/L	200 ng/L	[85]
	Ferrocene	DPV	miR-141	500 aM-50 nM	138 aM	[86]
Low dimensional carbon materials	SWCNT	DPV	Arsenite	0.5–10 ppb	0.5 ppb	[87]
	GO	DPV	DNA	----	500 pM	[63]
	MWCNT	Electrical resistance	HIgG	25–200 μg/mL	----	[68]

‘----’ Data not provided in the literature.

## Abbreviations

AFP	Alpha-fetoprotein
Ag NPs	Silver nanoparticles
ALP	Alkaline phosphatase
anti-tTG	Anti-tissue transglutaminase immunoglobulin A antibodies
ApoE	Apolipoprotein E
ATP	Adenosine triphosphate
Au NPs	Gold nanoparticles
BSA	Bovine serum albumin
CA125	Carcinoma antigen 125
CdS QDs	Cadmium sulfide quantum dots
CdSe@ZnS QDs	Cadmium selenide@Zinc sulfide quantum dots
CdTe QDs	Cadmium telluride quantum dots
CEA	Carcinoembryonic antigen
cTnI	Cardiac troponin I
cTnT	Cardiac troponin T
Cu NPs	Copper nanoparticles
CV	Cyclic voltammetry
CVs	Cyclic voltammograms
DNA	Deoxyribonucleic acid
DPV	Differential pulse voltammetry
DT-D	DT-Diaphorase
DμFD	Disposable microfluidic device
*E. coli*	*Escherichia coli*
ECL	Electrochemiluminescence
EIS	Electrochemical impedance spectroscopy
Fe_3_O_4_ NPs	Ferromagnetic nanoparticles
G6PDH	Glucose-6-phosphate dehydrogenase
GCE	Glassy carbon electrode
GIgG	Goat immunoglobulin G
GN	Graphene
GO NPs	Graphene oxide nanoparticles
GO	Graphene oxide
GOx	Glucose oxidase
GPDH	Glycerol-3-phosphate dehydrogenase
GSH	Glutathione
HgSe NPs	Mercury selenide nanoparticles
HIgG	Human Immunoglobulin G
HRP	Horseradish peroxidase
IgG	Immunoglobulin G
IL-6	Interleukin-6
Ir NPs	Iridium nanoparticles
IrO_2_ NPs	Iridium oxide nanoparticles
ITO	Indium tin oxide
LOx	Lactate oxidase
LSV	Linear sweep voltammetry
MBs	Magnetic beads
miR-141	MicroRNA 141
miR-21	MicroRNA 21
MoS_2_ NPs	Molybdenum disulfide nanoparticles
MSNs	Mesoporous silica nanoparticles
MSO	Mercury-specific oligonucleotide
MWCNT	Multi-walled carbon nanotubes
NPs	Nanoparticles
ORR	Oxygen reduction reaction
PAP	p-aminophenol
PAPG	p-aminophenyl galactopyranoside
PBS	Sodium phosphate buffer
PCB	Polychlorinated biphenyls
Pd NPs	Palladium nanoparticles
PDBE	Polybrominated diphenyl ethers
PEC	Photoelectrochemical
POC	Point-of-care
PSA	Prostate specific antigen
Pt NPs	Platinum nanoparticles
PTH	Parathyroid hormone
QDs	Quantum Dots
Rct	Charge transfer resistance
RIgG	Rabbit immunoglobulin G
*S. typhi*	*Salmonella typhimurium*
SARS	Severe acute respiratory syndrome
SEM	Scanning electron microscope
SPEs	Screen-printed electrodes
ssDNA	Single stranded DNA
SWASV	Square wave anodic stripping voltammetry
SWCNT	Single walled carbon nanotubes
SWSV	Square wave stripping voltammetry
SWV	Square wave voltammetry
TCID	Tissue culture infective dose
t-DNA	Transfer DNA
TEM	Transmission electron microscopy
TGA	Thioglycolic acid
TiO_2_ NPs	Titanium dioxide nanoparticles
TMB	3,3′,5,5′-Tetramethylbenzidine
XPS	X-ray photoelectron spectroscopy
ZnS NPs	Zinc sulfide nanoparticles
α-SYN	α-Synuclein
β-gal	β-galactosidase
